# Nonlinear deformation and localized failure of bacterial streamers in creeping flows

**DOI:** 10.1038/srep32204

**Published:** 2016-08-25

**Authors:** Ishita Biswas, Ranajay Ghosh, Mohtada Sadrzadeh, Aloke Kumar

**Affiliations:** 1 Department of Mechanical Engineering, University of Alberta, Edmonton, T6G 2G8, Canada; 2 Department of Mechanical and Aerospace Engineering, University of Central Florida, Orlando FL 32816, USA

## Abstract

We investigate the failure of bacterial floc mediated streamers in a microfluidic device in a creeping flow regime using both experimental observations and analytical modeling. The quantification of streamer deformation and failure behavior is possible due to the use of 200 nm fluorescent polystyrene beads which firmly embed in the extracellular polymeric substance (EPS) and act as tracers. The streamers, which form soon after the commencement of flow begin to deviate from an apparently quiescent fully formed state in spite of steady background flow and limited mass accretion indicating significant mechanical nonlinearity. This nonlinear behavior shows distinct phases of deformation with mutually different characteristic times and comes to an end with a distinct localized failure of the streamer far from the walls. We investigate this deformation and failure behavior for two separate bacterial strains and develop a simplified but nonlinear analytical model describing the experimentally observed instability phenomena assuming a necking route to instability. Our model leads to a power law relation between the critical strain at failure and the fluid velocity scale exhibiting excellent qualitative and quantitative agreeing with the experimental rupture behavior.

Bacterial streamers, which are microscopically slender filamentous aggregates primarily comprising of bacterial cells encased in matrix of self-secreted extra-cellular polymeric substances (EPS)^1^ and formed typically under sustained hydrodynamic flow. Bacterial streamers have been found to form in both turbulent flow conditions^2^,^3^ and even creeping flow conditions (Reynolds number (*Re*) ≪ 1)^4^,^5^. Streamer formation under low Reynolds number (*Re* < 1) conditions has recently attracted attention as they can have significant impact on the performance of filtration units^6^,^7^ and biomedical devices^4^,^8^,^9^. More importantly, their filamentous structure can extend significantly with flow^5^,^10^,^11^, thereby spanning several disconnected surfaces otherwise not easily possible for other similar aggregative modes of bacterial life such as flocs, pellicles or biofilms. This can make colonization rapid, pervasive and resistant to erosion with flow^4^,^12^. Therefore, a better understanding of deformation, failure and disintegration of streamers forming in low Reynolds number conditions is crucial from both physical and biological perspectives. Unfortunately, the literature on this subject is sparse and often lacks a deeper mechanistic exploration. For instance, Valiei *et al*.^11^ had reported observing failure of streamers in their experiments on a microfluidic device with micro-pillars. However, since the primary focus was formation of streamers, failure was not discussed in detail. Das and Kumar^13^ carried out a theoretical investigation on the formation and disintegration of biofilm streamers treating them as liquid jets in their terminal configuration. This assumption, while suitable for streamer formation time-scales (*t*_*s*_) of several hours, seems to be too restrictive in light of the subsequent and current experiments^12^ which indicate that streamers can also form at very short time-scales (*t*_*s*_~*seconds*) with significant initial and residual elasticity. The overall complexity of the material behavior of the streamers is due to the EPS which contains biological macromolecules whose unfolding and ‘flow’ can introduce highly nonlinear stress-strain relationships^14^,^15^, and the embedded, significantly stiffer biological cells^16^ forming a multiphase composite soft media. Thus, studying deformation and failure of streamers can be especially challenging since the complexity of the material constitution and formation process itself can be significantly reflected and amplified. Another challenge that plagues such studies is that as these streamers form in very small scale devices, and thus they do not accrue enough biomass to be subjected to *ex-situ* material characterization tests. Moreover, *ex-situ* measurements are usually very invasive for the delicate biomass. Hence, *in-situ* measurements and/or characterization methods must be developed.

In this paper, we report a quantitative study and *in-situ* observation of the ultimate failure and instability of bacterial streamers formed from bacterial flocs^12^ for two separate bacterial strains using a microfluidic platform. We observe specifically that although the streamer material is a complex composite soft material and remains tightly influenced by the nature and dynamics of the immersed fluid, a highly localized failure via necking is common and widespread in this type of system. This may be contrasted to the global nature of hydrodynamic instabilities which thus indicates that even up until the terminal stages, the streamers retains significant distinctions from purely viscous jets^13^. We also carry out analytical instability analysis assuming a localized failure on a simplified system incorporating mechanical nonlinearity, surface tension and fluidic loading which provides a power law scaling between the strain at failure and background flow which shows excellent qualitative and quantitative agreement with our experimental observations. Interestingly, this highly localized failure also ensures that a significant portion of the streamer, attached to the wall, is still preserved unlike global instability or shear failure at the wall. Thus, we find that streamer failure may not coincide with actual annihilation or erosion of streamers. To the best of our knowledge, this is the first study that reports a quantitative *in-situ* observation of the failure along with a continuum mechanics model for the same.

## Results

**Figure 1:**
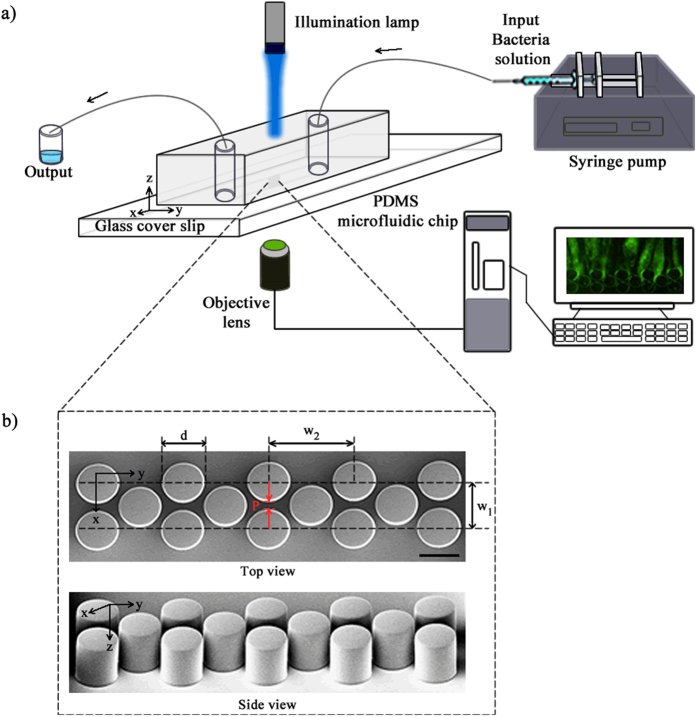
(**a**) Schematic of experimental set-up under pressure driven flow and (**b**) the SEM image of the micro pillars. The dimensions are *d* = 50 μm, *w*_*1*_ = 60 μm, *w*_*2*_ = 104 μm and *P* = 10 μm. The scale bar is 50 μm.

**Figure 2:**
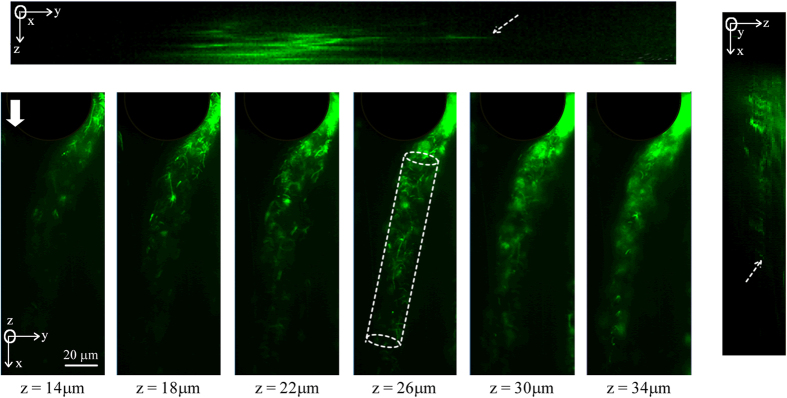
The geometry of one *P. fluorescens* streamer. Confocal sidebars shows *y-z* plane (top) and *z-x* plane (right) the thickness of one streamer through the height of the pillar. The approximate length of the streamer is 190 μm, width and thickness are 20 μm. White arrow shows the flow direction in the chip. The dashed white lines show the cross-section of the streamer in the *x-y* plane. The relatively similar cross sectional span and their uniformity through the depth of the streamer confirm a relatively cylindrical profile of the streamer away from the wall. The dashed arrows on the confocal sidebars show that the streamer does not come in contact with either the ceiling/floor of the device.

Our microfluidic device (Fig. 1) consisted of an array of PDMS micropillars in a staggered grid pattern. The micropillars had a diameter of (*d*) of 50 μm and were spaced 10 μm apart (*P*) (Fig. 1b). Bacterial flocs laden fluid was injected in the device using a syringe pump and the volumetric flow rate (*Q*) was maintained at a level such that the resultant flow in the device was in the creeping flow regime (*Re* ≪ 1). The velocity scale (*U*) in the device is defined by the relationship *U* = *Q*/*W* × *h*_*c*_. Bacterial flocs laden flow in the device led to the rapid formation of bacterial streamers. Such a mode of streamer formation has already been studied by Hassanpourfard *et al*.^12^ which showed that bacterial flocs could adhere to micropillar walls and get rapidly sheared by background flow to form streamers. In an earlier publication, Hassanpourfard *et al*.^12^ had studied the inception of streamers from flocs and found nucleating streamers to be dominated by large recoverable strains indicating significant elasticity. In our work we employed this floc-mediated route to create streamers for two separate bacterial strains – (i) a green fluorescent protein (GFP) expressing *Pseudomonas fluorescens* strain and (ii) *Pseudomonas aeruginosa* (see Materials and Methods). These streamers typically have a cylindrical geometry with large aspect ratio. This can be seen in Fig. 2, which shows a *P. fluorescens* streamer imaged using confocal microscopy. The confocal sidebars in Fig. 2 also show that the section of interest of the streamer does not come in contact with either the ceiling/floor of the device.

**Figure 3:**
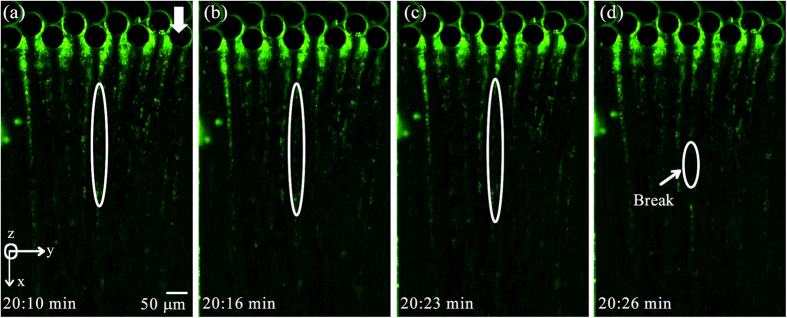
Observation of instability and failure in *P. fluorescens* streamers under fluorescence imaging using Green filter cube at *U* = *8.92* × *10*^*−4*^ m/s. (**a**–**c**) Shows the stretching of one streamer with time and final breaking point shows in (**d**). The arrow showing flow is aligned to the *x*-direction of the chip.

In the current experiments our focus is on observing nonlinear behavior including instabilities and failure of streamers. Figure 3 depicts failure in a streamer for a *P. fluorescens* streamer formed in our device after approximately 20 minutes of starting the experiment. The corresponding velocity scale and Reynolds number for the experiments were *U* = 8.92 × 10^−4^ m/s and *Re*~10^−3^. A similar behavior is observed for *P. aeruginosa*. These experiments which clearly show the evidence of far from wall failure and instability are nevertheless less useful in quantifying the failure process due to the invisibility of the EPS. The bacteria themselves although clearly visible are a poor proxy for quantification due to their relatively non-uniform and sparse distribution in the EPS. In order to alleviate this problem, we repeat these experiments with 200 nm red fluorescent amine-coated polystyrene micro-spheres, which are of different color, much smaller and numerous thereby making quantification and visualization of instability much easier and more accurate. We found relatively dilute concentration of approximately 0.009% (v/v) of nanoparticles compared to bacteria which was about 0.4% (v/v) (See Supplementary Information for volume fraction calculations). Since the Young’s modulus of bacterial cells (~*O*(10^2^ MPa))^17^ and polystyrene microspheres (~*O*(10^3^ MPa))^18^ are comparable, and they themselves undergo no noticeable deformation, this ensures little contribution of beads to the overall mechanical behavior of the streamer biomass itself.

**Figure 4:**
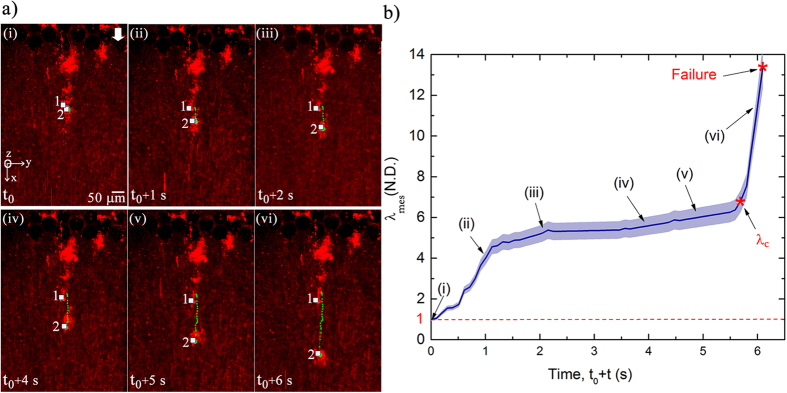
(**a**) Temporal behavior of two Lagrangian points (‘1’ & ‘2’), which lie inside a *P. fluorescens* streamer, culminating in the failure of streamer. Other pertinent parameters are *U* = *8.9* × *10*^*−4*^ m/s and *t*_*0*_ = 30.34 minute. (**b**) Stretch ratio for the same streamer as a function of time. The blue shaded region denotes the estimated error envelope. Data points corresponding to the experimental conditions (i–vi) are depicted on the curve. Stretch ratio is not computed once failure occurs in the streamer. Note that the blue shaded region denotes the estimated 4% tracking error envelope applicable for any observed streamer (See Supplementary Information for uncertainty estimates).

**Figure 5:**
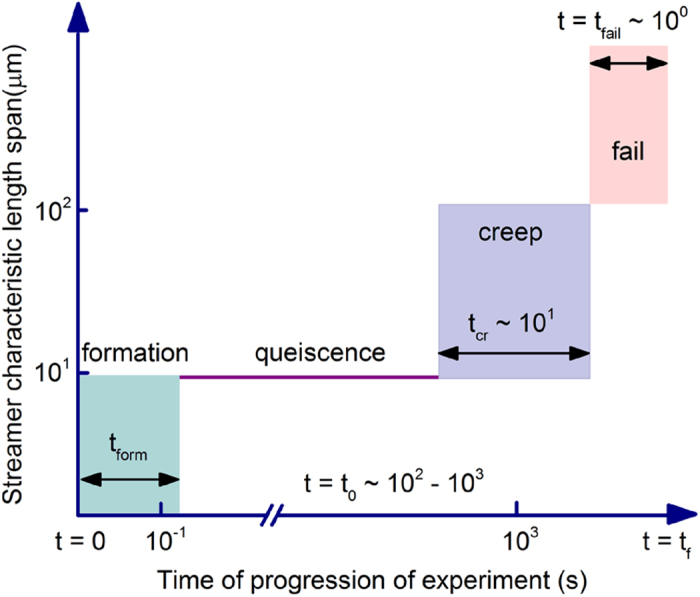
Schematic illustration of space-time scales corresponding to regimes observed in the experiments.

Utilizing this technique, we depict the visualization and quantification of a *P. fluorescens* streamer undergoing axial failure in Fig. 4, where embedded particles are used to illuminate the EPS. The background velocity scale for the experiment was kept constant at *U* = 8.92 × 10^−4^ m/s during this experiment. These observations indicated that the streamer deformation clearly occurs in three distinct phases, (see Supplementary Movie 1). The first - formation stage occurs almost as soon (*t*_*form*_) as the flow stabilizes, which is typically in seconds and in agreement with earlier streamer formation experiments on similar systems^12^. The streamers thus formed continue to retain their shape and size, remaining apparently static against a steady background flow till about time *t*_0_ ≫ *t*_*form*_ when deformation begins again. The time between *t*_*form*_ and *t*_0_ where the streamer formation is complete but the next stage of deformation is not yet perceptible can last for several minutes. The nature of this quiescence is as of yet unclear and may simply be another creep stage with extremely small strain rate or a period of microstructural rearrangement before perceptible creep sets in. In any case, we are interested in quantifying the deformation which was observed only after *t*_0_. After *t*_0_, which can last for several minutes after the initiation of the experiment, streamers begin to deform perceptibly, resembling a creep type deformation over a much larger time scale (*t*_*cr*_ ≫ *t*_*form*_). This creep stage ends rather abruptly through a short region of increasing deformation culminating in a distinct sharply defined instability after which the streamer suffers terminal failure through a final catastrophic large deformation stage occurring over a much smaller time scale (*t*_*fail*_ ≪ *t*_*cr*_). The different space-time scales of the observed deformation regimes, which are of the same order for both bacterial strains, are schematically illustrated in Fig. 5.

A better quantification of this phenomenon is possible due to the embedded particles mentioned earlier. Two such particles which lay on either side of the localized failure zone were specifically tracked in the video from the beginning of the creep phase (time *t*_0_) to measure the temporal evolution of deformation (Fig. 4a). The deformation is quantified using a non-dimensional measured ‘stretch ratio’ defined as λ_*mes*_ = |*dx*|/|*dX*|, where |*dX*| is the distance between the two points at time *t*_0_ and |*dx*| is the distance between the two points at any given time *t* (>*t*_0_). In this context, it is useful to recall that there are several ways to quantify deformation. Any general deformation can be decomposed into a rotation and a stretch (Polar decomposition theorem^19^. Thus stretch is a primitive form of deformation quantification. Strains are functions of stretches and can be of many different types (e.g. Engineering, Cauchy-Green, Green-Lagrange, Hencky etc.) depending on the choice of the problem. Therefore, we have used stretch as a fundamental and intuitive measure of deformation for this problem.

Figure 4b depicts the time-evolution of *λ*_*mes*_ for a couplet on a particular *P. fluorescens* streamer. It is important to recall that *λ*_*mes*_ = 1 corresponds to the time when the streamer formation and initial deformation in response to the fluidic loading is complete and thus the beginning of the creep stage. In this regards, observing that the background flow is steady with little mass addition in this phase, Fig. 4a, and the fact that creep is seen to occur over a much shorter time scale than typical bacterial reproduction time-scale 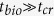 (~20 min)^20^, we conclude that the creep type deformation has origins in the material constitution. Within the creep stage, we find three distinct deformation regimes beginning first with a rather rapid strain rate regime, which then decreases significantly indicating substantial hardening of the material, stabilizing to almost a constant strain rate. This continues for some time till the strain rate begins to increase again and the streamer deformation accelerates, transitioning after a critical stretch ratio *λ*_*c*_ into the terminal failure stage, mentioned earlier. The ultimate failure process observed through microscope shows significant localization, with the streamer structure left almost entirely intact at either side of instability resembling a necking route to failure of solids under axial loading. This failure behavior is observed in both bacterial strains suggesting a mechanistic origin of the instability.

**Figure 6:**
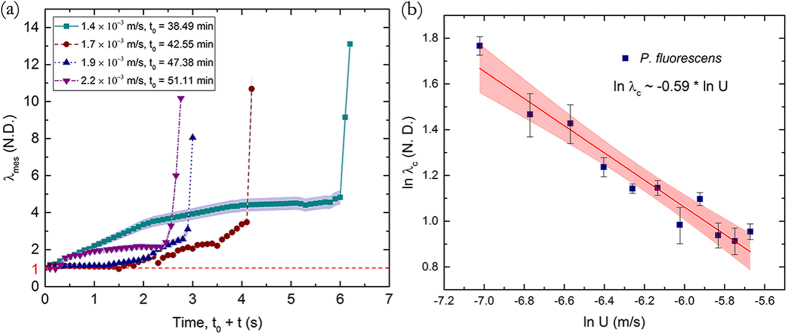
(**a**) Stretching of *P. fluorescens* streamers with time at different background flow velocity scale (*U*) and *t*_*0*_. Note that the blue shaded region denotes the estimated 4% tracking error envelope applicable for any observed streamer (See Supplementary Information for uncertainty analysis) (**b**) ln (*λ*_*c*_) for different flow rates, shows a linear behavior with ln (*U*). The blue squares represent experimental data and the red line represents a linear regression fit. The *R*^*2*^ value corresponding to the regression fit is greater than 0.9. The red envelope depicts the 95% confidence interval for linear regression.

**Figure 7:**
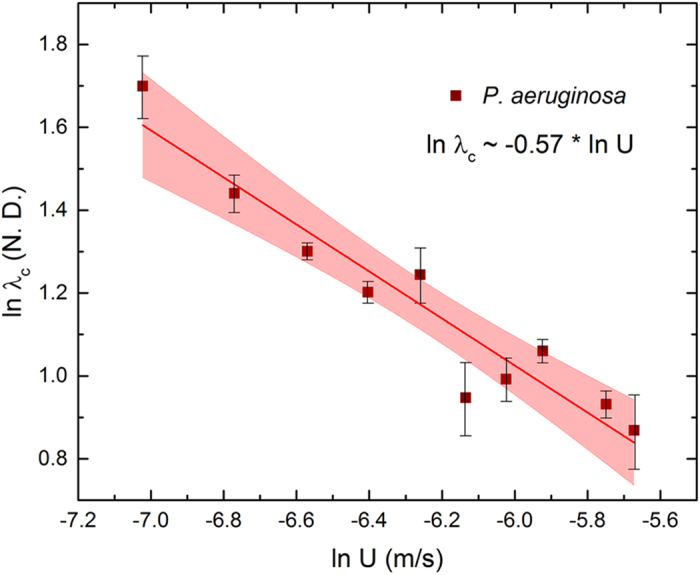
ln (*λ*_*c*_) for different flow rates for for *P. aeruginosa* streamers show a similar scaling with ln (*U*). The brown squares represent experimental data and the red line represents a linear regression fit.

Note that within these broad stages of deformation, some irregularities are possible for some streamers such as temporary secession of creep and then sudden resumption due to local rearrangement of the micro or meso-structure of the highly complex streamer constitution. We also note that the setup used in these experiments can result in multiple streamers being formed at various locations and at different times. However, when the temporal origins are normalized and each streamer tracked with particle couplets using the method described earlier, we observe broadly similar distinctive creep regimes which lasted for similar time scales (although with different deformation magnitudes for a given regime) and ended with similar critical stretch ratios across streamers considering the enormous variation possible in their material composition and shape (See Supplementary Information Fig. SF2 for a sample set of streamers). This broad generality is reinforced in the next figure, Fig. 6a where we plot the creep response of *P. fluorescens* streamers under different flow velocities and notice again very similar deformation regimes (see Supplementary Information for Uncertainty Analysis). However, the duration of the regimes vary considerably with the flow velocity. More interestingly, we find that the critical axial stretch ratio at failure steadily decreases with increasing flow, Fig. 6b, defining thereby a clear monotonic decreasing rupture envelope. This is in clear contrast to liquid jet breakup which indicates a monotonic increasing envelope^13^,^21^. More interestingly, we find a clear power law scaling of the failure stretch rate with flow, *λ*_*c*_~*U*^*α*^ where *α* = −0.59 ± 0.052, Fig. 6b. When these experiments were repeated for *P. aeruginosa* we were able to recover a very similar power law with *α* = −0.57 ± 0.060 (Fig. 7). Thus, we see that the scaling relationship *λ*_*c*_~*U*^*α*^ appears to be a general biophysical phenomena with an average exponent *α* ≈ −0.58.

In order to extract further insights from these experimental observations, we take recourse to an analytical stability analysis of a simplified version of this structure. To this end we first recall that the measurements of axial stretch ratios were taken by tracing the tracking couplets back in time till a creeping type deformation begins. It is typical to assume that the total stretch *λ* can be multiplicatively decomposed into elastic *λ*_*e*_ and a creep component *λ*_*cr*_^22^. Furthermore, we assume that in light of extreme deformation suffered by the initial floc which precipitates the streamer formation^12^, the limiting chain length extension^23^,^24^,^25^ of the polymeric material dominating the streamer is reached at the end of the elastic phase. This behavior is consistent with preliminary characterization of biofilm materials which show pronounced strain stiffening^16^. With this simplification, we can assume that the experimentally reported stretch ratios in this paper are purely due to creep with elasticity preceding the origin of the plots. This split in the regimes would mean that the experimentally reported stretch ratios in Figs 4b and 6a are essentially *λ*_*cr*_ = *λ*/*λ*_*e*_ ≈ *λ*_*mes*_ and that the incremental deformation measured are entirely due to creep. Now assuming uniform conditions inside the streamer, the logarithmic axial creep strain 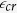 and strain rate 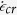 can be written as:


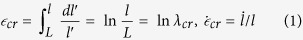


where *L* is the length of the streamer after the elastic deformation (formation stage) is complete and *l* is the current length. Imposing isochoric deformation in inelastic regime and assuming a cylindrical geometry of the specimen throughout deformation (akin to long wavelength defect approximation for axial tensile stability analysis^26^,^27^), we relate the current geometry to the reference geometry corresponding to the end of formation stage. To this end if the reference cylinder had a radius *R* (and length L) which is deformed in the inelastic regime into another cylinder of radius *r* (and length *l*), the reference slenderness ratio Ω = *L*/*R* can be related to the current slenderness ratio *ω* = *l*/*r* as:


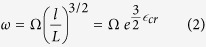


Note that for a linear elastic isotropic solid, a total of two independent material constant are needed for complete characterization. These are the Young’s moldulus (*E*) and Poisson’s ratio (ν). The Poisson’s ratio is the ratio of the strains in the lateral to longitudinal direction. If the constraint of isochoric deformation (sometimes called incompressibility) is imposed on the system, one can obtain ν = 0.5^28^. On the other hand, the inelastic regime is often assumed isochoric independently from the elastic case for a large number of materials and the physical origin of this assumption can be found in classical treatises in plasticity^29^,^30^. Thus in this case, in the inelastic regime the constraint of isochoric deformation and shape preservation dictates the evolution of the slenderness ratio.

**Figure 8:**
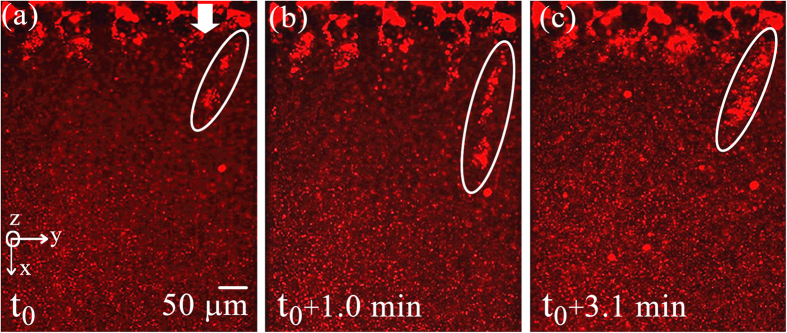
Stretching and relaxation of a *P. fluorescens* streamer as flow rate is increased (*a* → *b*) and subsequently decreased (*b *→ *c*).

Using confocal microcopy we confirm a reasonably cylindrical geometry of the specimen away from the walls along with a high slenderness ratio (length to radius ratio ≈ 10), Fig. 2, which is imaged using only green fluorescence where only *P. fluorescens* bacterial cells are visible with both the encapsulating EPS and liquid media appearing dark. Now, if we assume that the surrounding fluid only exerts traction and does not lead to significant mass addition, the streamer and the force field can thus be assumed to be a closed system exchanging only heat with a thermal reservoir to maintain isothermal conditions. Using the internal variable framework incorporating internal dissipation^31^,^32^, instability is then said to occur when perturbation to neighboring states can result in decrease in total free energy *G*. In other words, for instability:


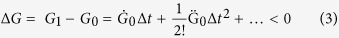


where Δ*t* is the perturbation time of interest. Now note that due to the stationary nature of equilibrium, 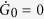 and thus it is the sign of the second derivative which will determine the stability of equilibrium. Specifically, the instability conditions can be obtained through the equation 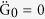 which denotes the point of transition from stability to instability. To evaluate the condition of instability we first write the free energy rate for this system in the current configuration:


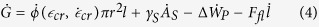


where *φ* is the dissipation density function which can in general depend on the strain and strain rate, *γ*_*S*_ is the surface tension (assumed uniform and without any Marangoni effects), *A*_*s*_ is the rate of change of surface area, Δ*W*_*p*_ is the rate of work done by the pressure difference between the inside and outside of the streamer and *F*_*fl*_ is axial the fluidic traction force. In subsequent calculations, we will neglect end area of the cylinder in our energy calculations due to the observed high slenderness ratio (Ω, *ω* ≫ 1). The high slenderness ratio when combined with the low Reynolds number flow also allows us to use the slender body approximation for our fluidic force on the cylinder leading to^33^ *F*_*fl*_ = *CπμU*/In *ω* where *μ* is the viscosity of the fluid, *U* is the fluid velocity scale and *C* is an appropriate scaling constant. Next, it can be shown that the free energy rate in Eq. (4) can be written in terms of per unit volume as (See Supplementary Information for detailed derivation):


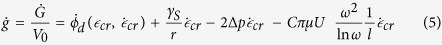


where *V*_0_ is the constant reference volume. Before evaluating second derivative, we first note from Fig. 4b that in our problem the strain rate variation before the onset of instability has a much higher Deborah number 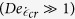 with respect to the time scale of instability precipitation and can thus be assumed to be frozen^31^ for our current calculation. On the other hand, the pressure differential has a much lower Deborah number (*De*_Δ*p*_ ≪ 1) due to relatively free passage of water throughout the streamer and thus assumed to be nearly equilibrated^31^ for the time scale of our calculation. With these assumptions, we have the following instability condition from the vanishing second derivative of the free energy density (See Supplementary Information for detailed derivation):


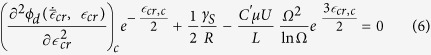


where 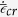 denotes the frozen strain rate for our calculation, *C*′ is a positive dimensionless constant and subscript *c* indicates the state at the point of instability. Next, we develop a simple model to quantify the dissipation potential which should be consistent with the observed strain hardening of the streamer material. To this end, we neglect poroelasticity as a source of creep since we already assumed that fluid can flow in and out of the streamer creating a drained condition in the time scale of interest. On the other hand, a phase change into a liquid phase is also ruled out through direct observation of elastic recoil even after the onset of terminal instability using a separate quenching experiment where the flow was suddenly stopped thus removing external loading on the streamer, Fig. 8. The creep dissipation behavior for the streamer material is currently unquantified. However, for this work, analogous to hyperelastic strain energy density functions, we would assume that the dissipation density function depends on the invariants of the left Cauchy-Green creep strain tensor^34^. For simplicity we postulate a linear dependence of the dissipation potential *ϕ*_*d*_ on the first invariant again drawing analogy from simple hyperelastic strain energy density functionals giving us the following functional form:


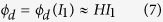


where *H* is the hardening constant which can in general depend on stain rate and *I*_1_ is the first invariant of the strain. Note that 
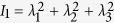
 where *λ*_1_, *λ*_2_, *λ*_3_ are three principal stretches of the streamer. Note that our previous study^12^ on the subject highlighted both the strain rate dependence as well as significant elastic component of deformation in streamers under uniaxial loading even in large deformation. In this problem we assume that the significant inelastic deformation is perceptible (yield) after the streamers have exhausted their elastic limit of deformation. Thus any more deformation would arise from purely inelastic sources. In other words, further work done on the system would no longer be stored as elastic energy but need to be dissipated, quantified by the dissipation potential. The strain rate effects (viscous effects), on the other hand, which were present even in the pre-yield phase would still be perceptible in the dissipation regime since their origin is distinct from the purely elastic component. However, in the plateau region (see Fig. 4b), the strain rate is roughly constant till the instability begins to precipitate. Thus the strain rate components in the material models would simply be constants, which have been absorbed in the dissipation potential postulated in the problem. Assuming uniaxial tensile stretching for the streamer in the creep regime, with axisymmetry and incompressibility, we get the three principal stretches as 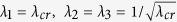. Thus the dissipation potential becomes from Eq. (7). 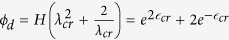. Now neglecting the negative exponential in *ϕ*_*d*_ we have 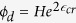 which when plugged into Eq. (6) gives us:


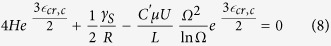


Now note that due the limiting chain extensibility argument in the elastic regime, Ω, *L* are determined by the limiting chain behavior of the streamer material. In general, the limiting chain extensibility is not a material constant but depends on the type of loading^23^, however since the loading can be assumed to be more or less similar in the current experiment, it becomes a de facto material constant. Moreover, since the streamers have been shown to originate from the deformation of flocs, whose dimensions are roughly similar^12^, we will assume that the dimension of the streamer at the end of elastic deformation remains fairly constant irrespective of the flow rate. If we denote the limiting chain material constant as *J*_*m*_ such as the one used in the widely employed Gent hyperelastic^35^ or a similar constant from the Van der Waals polymer model^36^, we can rewrite Eq. (8) as:


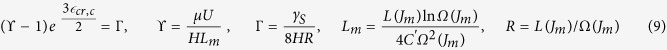


Now let us assume that ϒ ≫ 1, in other words hardening is sufficiently small or limiting chain extensibility is sufficiently large. With these assumptions, taking logarithms both sides yields 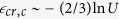. This scaling is in excellent agreement with our experimental observations described earlier which indicates 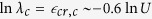 where *λ*_*c*_ is the measured stretch ratio at instability, Figs 6b and 7. Note that in the high ϒ limit, the actual value of *H* does not come in the final scaling since it cancels on both sides of Eq. (9), leaving only the elastic and surface tension properties along with fluid viscosity, i.e., 
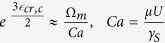
 is the capillary number and Ω_*m*_ = (*L*_*m*_)/(*R*). This relatively weak dependence on inelastic parameter somewhat narrows down the bandwidth of variation of this mode of streamer instability at a given flow and along with the inherent slender geometry may contribute to the observed generality of instability behavior across streamers in spite of numerous well known complexities and variations in the streamer constitution.

## Discussion

In this paper, we studied the deformation and failure behavior of bacterial streamers for two separate bacterial strains by passing a floc laden flow through a specially designed microfluidic device. The flow corresponded to very low Reynolds numbers and was sufficient to germinate streamers over time scales and configuration in agreement with prior experiments on streamer formation. However, we found that the apparent stability of the formed streamers transitioned slowly into complex creep like deformation regimes with their own characteristic time scales in spite of stable background flow conditions. The final stages of deformation were characterized by high strains over relatively small time scales leading to failure of the streamers. However, unlike previously believed fluid like models which could account for the creep as well as terminal failure of the streamers through a global hydrodynamic instability, we discovered highly localized failure of the streamers in our current experiments. Significant streamer recoil observed after the flow was stopped at the onset of instability confirmed residual elasticity. More interestingly, the distinct deformation regimes and the failure behavior of the streamers were broadly exhibited by different streamers at different flow conditions in spite of the highly complex and heterogeneous nature of the streamers themselves. That this behavior was common between streamers of two separate bacterial strains indicated a mechanistic route despite the system being biophysical in origin. We developed a simplified but nonlinear mechanical model incorporating inelasticity and surface tension to account for the failure behavior assuming necking failure of the streamers. Our model based on these assumptions faired remarkably well against the rupture scaling obtained from our experiments. Interestingly, this mode of failure was found to be entirely different from shear failure at wall or global hydrodynamic instability. Last but not the least, since this mode of failure does not lead to a global disintegration of the streamer, its effect on biofouling could be different from other modes of failure.

## Materials and Methods

### Design and microchip fabrication

A polydimethylsiloxane (PDMS) on glass microfluidic device was constructed using conventional photolithography process (Fig. 1a). First a 4′′ silicon master mold was prepared from a CAD drawing. Then the mold was further used to make the microfluidic device (chip). PDMS (Sylgard 184, Dow Corning, NY, USA) was used to prepare the chips. We considered PDMS because of its optically transparency, nontoxic nature and ease of fabricating a smooth and nonpolar surface. Fabrication of straight and smooth pillars walls in the channel was confirmed by scanning electron microscopy (SEM) imaging (Fig. 1b). The height of the pillars (*h*_*c*_) is 50 μm in *z* direction and the total width (*W*) of the chip is 436 μm. PDMS and glass slides were exposed to the oxygen plasma for 30 s, followed by bonding of PDMS to glass. The device (PDMS and glass coverslip) was then annealed at 70 °C for 10 minute to seal the channel.

### Bacterial solution preparation

We used *Pseudomonas fluorescens* CHA0 (wild type)^37^ and *Pseudomonas aeruginosa* MPAO1 (wild type) (*P. aeruginosa* Mutant Library - University of Washington) bacterium for this study. The bacteria strain from −80 °C collection was incubated overnight in the Luria – Bertani (LB) agar plate at 30 °C. One single colony was taken from the agar plate and poured in Luria – Bertani (LB) broth. *P. fluorescens* was incubated in this media for 14 hours in shaking incubator (Fisher Scientific, Canada) at 30 °C and 150 rpm and *P. aeruginosa* was incubated in this media for 1 hour and 20 minute in shaking incubator at 37 °C and 150 rpm. Prior to injection into the microfluidic device 200 nm red fluorescent amine-coated polystyrene particles were mixed with the bacteria solution in volume percent concentration of 0.04% (v/v).

### Microscopy

The microfluidic device was placed on the stage of an inverted optical microscope (Nikon Eclipse Ti) and confocal microscope (Olympus IX83) as shown in Fig. 1a. A syringe pump (Harvard Apparatus, Canada) was used to inject the solution into the microfluidic device. Epi-florescence capability of the microscope was utilized to image using either a GFP Long-pass Green filter cube or Texas Red filter cube (Nikon & Olympus). Tracking was performed by using the measurement tracking module of the NIS-Element AR software interface.

## Additional Information

**How to cite this article**: Biswas, I. *et al*. Nonlinear deformation and localized failure of bacterial streamers in creeping flows. *Sci. Rep.* **6**, 32204; doi: 10.1038/srep32204 (2016).

## Supplementary Material

Supplementary Movie 1

Supplementary Information
