# Supplementary material for: Nonlinear deformation and localized failure of bacterial streamers in creeping flows
Source: Sci Rep. 2016 Aug 25;6:32204. doi: 10.1038/srep32204 (PMC4997318; doi:10.1038/srep32204)
Supplement: Supplementary Information [file srep32204-s2.pdf]

Supplementary Information for

Nonlinear deformation and localized failure in bacterial streamers in creeping flows

Ishita Biswas[a], Ranajay Ghosh[b], Mohtada Sadrzadeh and Aloke Kumar[a]*

[a]Department of Mechanical Engineering, University of Alberta, Edmonton, Canada

[b]Department of Mechanical and Aerospace Engineering, University of Central Florida, Orlando FL 32816, USA

Corresponding author email: aloke.kumar@ualberta.ca

Volume Fraction Calculation

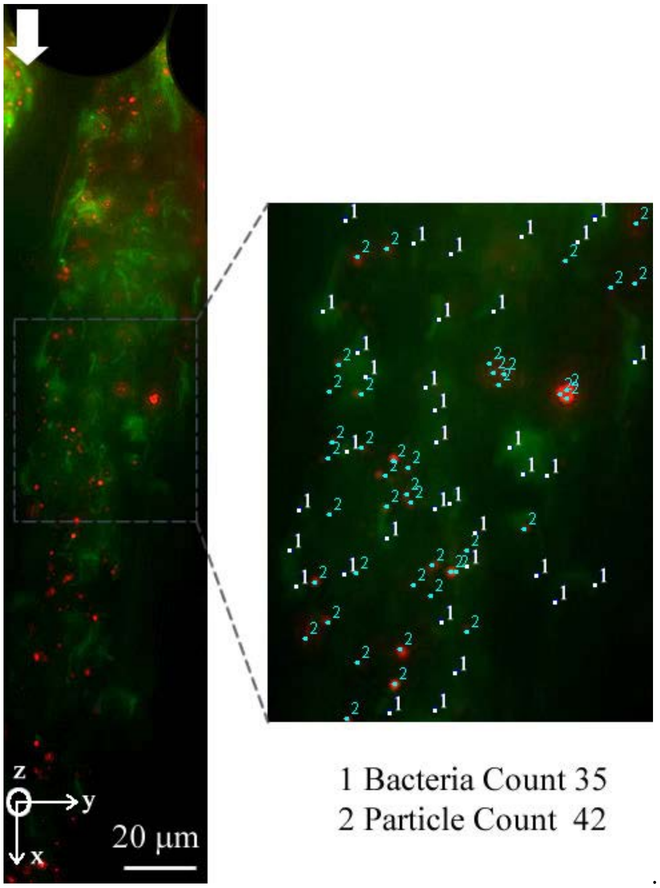

Figure SF1: Bacteria and particle count in one *P. fluorescens* streamer's failure zone (dotted box). The volume of zoomed picture is calculated by multiplying the area of the picture with depth of field and is approximately 2000 μm^3. Bacteria cell is cylindrical and particles are spherical in geometry. Diameter of bacteria and particle are 0.2 μm and the height/ length of one bacteria is 8 μm. Volume of one bacteria is 0.25 μm^3 and one particle is 4.19 $\times 10^{-3}$ μm^3.

Estimating Experimental Uncertainty

Two separate sources of experimental uncertainty were identified. The first is the repeatability error accounting for the heterogeneity of the biomass itself. Due to the very nature of this error, it has to be evaluated by statistical means (i.e. from a number of repeated observations). For each of 2 cases, the experiments were repeated 2-4 times for all flow rates to yield relative uncertainty estimates. For example, for the *P. fluorescens* for the flow velocity (U=8.92 $\times$ 10^{-4} m/s), the experiment was repeated 3 times thus yielding relative uncertainty for each U (Fig. SF2). Table ST1 provides the complete list of repetitions for each case. Let this uncertainty be denoted by δ_{repeat}. The second source of uncertainty resulted from an error in the tracking process itself. This error could be estimated by visually determining the uncertainty in tracking the Lagrangian points and the maximum error was estimated to be approximately 4%. This determines the error

envelope for a single tracking of couplets. Let this uncertainty be denoted by $\delta_{tracking}$. The final error envelope (δ) for critical stretch ratio is given by: $\delta = \sqrt{\delta_{repeat}^2 + \delta_{tracking}^2}$. Error bars in Figs. 6b and 7 represent δ.

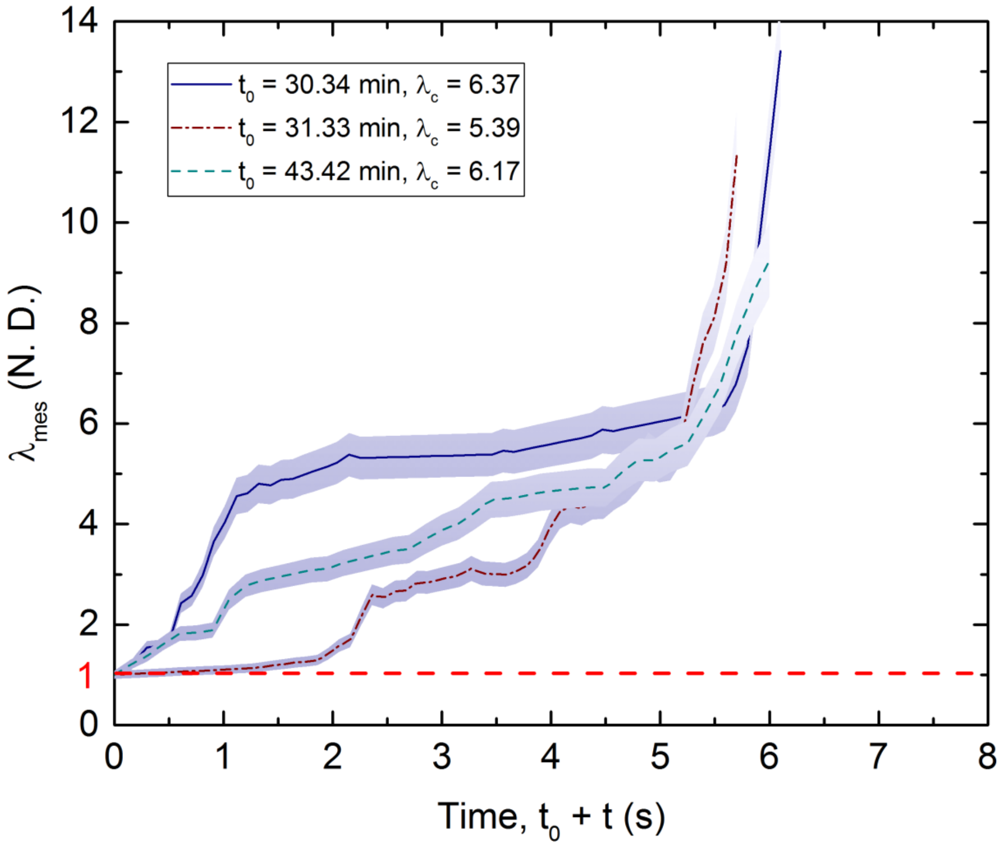

Figure SF2: Stretch ratio for three different failure events of *P. fluorescens* streamers at a constant background velocity of $U = 8.92 \times 10^{-4}$ m/s. Legend shows the t_0 and critical stretch ratio for the three different cases.

Table ST1: Repeatability data for finding different λ_c for different flow rates for the two cases.

**Parameters**	**λ_C (N. D.)**	
**Velocity, U, m/s**	***P. fluorescens***	***P. aeruginosa***
**8.92 × 10^{-04}**	6.00	5.83
	5.39	5.74
	6.17	4.29
	-	6.03
**1.15 × 10^{-03}**	3.35	3.95
	4.87	4.11
	4.04	4.60
	5.10	-
**1.40 × 10^{-03}**	4.82	3.66
	4.23	3.69
	3.46	-
**1.66 × 10^{-03}**	3.48	3.35
	3.54	3.25
	3.09	3.23
	3.67	3.47
**1.91 × 10^{-03}**	3.11	3.16
	3.14	3.78
	3.16	-
**2.17 × 10^{-03}**	3.29	3.09
	2.97	2.33
	3.17	2.31
**2.42 × 10^{-03}**	3.09	2.88
	2.67	2.51
	2.27	-
**2.68 × 10^{-03}**	3.02	2.76
	2.85	2.93
	3.14	2.83
	2.98	3.02
**2.93 × 10^{-03}**	2.82	-
	2.30	
	2.55	
**3.19 × 10^{-03}**	2.68	2.40
	2.61	2.57
	2.18	2.65
**3.44 × 10^{-03}**	2.40	1.99
	2.67	2.82
	2.70	2.34

Streamer Instability Calculations

Kinematics

The streamer is assumed to be slender cylindrical body (long wavelength defect approximation) throughout till the onset of instability. In the reference and current configuration, the radius of the cylinder are R, r and length L, l respectively. We also assume the streamer to have uniform behavior throughout its material volume except at the far field boundaries before the onset of instability.

We also assume that streamer deformation is purely inelastic after the elastic limit is reached and to remain in this state till the onset of instability. We assume that inelastic deformation is isochoric and describe the geometry of the current streamer (right cylinder) with radius r and length l. Thus, in the rate form we arrive at the following relationship:

$$2\frac{\dot{r}}{r} + \frac{\dot{l}}{l} = 0 \qquad \text{(S1)}$$

Now since the deformation after elastic limit is purely inelastic, we define the axial creep strain as $\delta\epsilon_{cr} = \delta l/l$. Integrating it from the elastic limit where the dimensions of the cylinder are assumed to be $R \times L$ to current configuration gives us the following logarithmic inelastic strain:

$$\epsilon_{cr} = \int_L^l \frac{dl\prime}{l\prime} = ln\frac{l}{L} = ln\,\lambda_{cr} \;\; , \;\; \dot{\epsilon}_{cr} = \dot{l}/l \qquad \text{(S2)}$$

where λ_{cr} is the creep stretch. We will refer to the elastic limit configuration to be the reference configuration for the purpose of this analysis. Now denoting the aspect ratio in the current configuration to be $\omega = l/r$ and the reference configuration to be $\Omega = L/R$, we can relate them using incompressibility:

$$\omega = \Omega\left(\frac{l}{L}\right)^{3/2} = \Omega\, e^{\frac{3}{2}\epsilon_{cr}} \qquad \text{(S3)}$$

Thus this also shows that $l = \omega^{2/3}\Omega^{-2/3}\,L$ and $r = R\Omega^{1/3}\omega^{-1/3}$. In the rate form Eq. (S3) becomes

$$\dot{\omega} = \frac{3}{2}\omega\,\dot{\epsilon}_{cr} \qquad \text{(S4)}$$

Mechanics

We first write the free energy rate for this system in the current configuration:

$$\dot{G} = \dot{\phi}(\epsilon_{cr}, \dot{\epsilon}_{cr})\pi r^2 l + \gamma_S \dot{A}_S - \Delta\dot{W}_P - F_{fl}\dot{l} \qquad \text{(S5)}$$

where ϕ is the dissipation density function which can in general depend on the strain and strain rate, γ_S is the surface tension (assumed uniform and without any gradient Marangoni effects), $\dot{A}_S$ is the rate of change of surface area, $\Delta\dot{W}_P$ is the rate of work done by the pressure difference between the inside and outside of the streamer and F_{fl} is axial the fluidic traction force. We can write the rate of change of surface area as:

$$\dot{A}_S = \frac{d}{dt}(2\pi rl + \pi r^2) \; = 2\pi rl\left(\frac{\dot{r}}{r} + \frac{\dot{l}}{l} + \frac{\dot{r}}{l}\right) \approx \pi r\dot{l} = \pi rl\dot{\epsilon}_{cr}, \;\; \omega \gg 1 \qquad \text{(S6)}$$

For the rate of pressure work, we have:

$$\dot{W}^{pr} = \Delta p 2\pi rl\,\dot{r} - \Delta p\pi r^2\dot{l} = \Delta p\pi r^2 l\left(\frac{2\dot{r}}{r} - \frac{\dot{l}}{l}\right) = -2\Delta p\pi r^2 l\,\dot{\epsilon}_{cr}$$

where $\Delta p = p_0^{ex} - p_0^{in}$ is the pressure differential between outside p_0^{ex} and inside pressure p_0^{in}.

Now note that from the slender body approximation of resistive flow theory we have, $F_{fl} = C\pi\mu U/\ln\omega$ and thus we get for the fluidic work rate, $F_{fl}\dot{l} = \frac{C\pi U l}{\ln l/r}\,\dot{l} = \frac{C\pi U l^2}{\ln\omega}\,\dot{\epsilon}_{cr}$

Using these expressions and then dividing Eq. (S-1) by the current volume $\pi r^2 l$ we have:

$$\dot{g} = \dot{\phi}_d(\epsilon_{cr}, \dot{\epsilon}_{cr}) + \frac{\gamma_S}{r}\dot{\epsilon}_{cr} - 2\Delta p\dot{\epsilon}_{cr} - C\pi\mu U\ \frac{\omega^2}{\ln\omega}\frac{1}{l}\dot{\epsilon}_{cr} \tag{S7}$$

Using the time scale approximations and the isochoric nature of the deformation (refer to the main body of the paper), the second time derivative of Eq. (S7) leads to

$$\begin{aligned}
\ddot{g} &= \ddot{\phi}_d(\epsilon_{cr}, \dot{\epsilon}_{cr}) - \frac{\gamma_S}{r^2}\ \dot{r}\dot{\epsilon}_{cr} - C\mu U\frac{d}{dt}\left(\frac{\omega^2}{\ln\omega}\frac{1}{l}\right)\dot{\epsilon}_{cr} \\
&= \ddot{\phi}_d(\epsilon_{cr}, \dot{\epsilon}_{cr}) + \frac{1}{2}R^{-1}\Omega^{-\frac{1}{3}}\omega^{\frac{1}{3}}\gamma_S\ \dot{\epsilon}_{cr}\dot{\epsilon}_{cr} - C\mu U\frac{d}{dt}\left(\frac{\omega^2}{\ln\omega}\ \omega^{-\frac{2}{3}}\Omega^{\frac{2}{3}}\,L^{-1}\right)\dot{\epsilon}_{cr} \\
&= \ddot{\phi}_d(\epsilon_{cr}, \dot{\epsilon}_{cr}) + \frac{1}{2}R^{-1}\Omega^{-\frac{1}{3}}\omega^{\frac{1}{3}}\gamma_S\ \dot{\epsilon}_{cr}\dot{\epsilon}_{cr} - C\mu U\ \frac{d}{dt}\left(\frac{\omega^{\frac{4}{3}}}{ln\,\omega}\ \Omega^{\frac{2}{3}}\,L^{-1}\right)\dot{\epsilon}_{cr} \\
&= \ddot{\phi}_d(\epsilon_{cr}, \dot{\epsilon}_{cr}) + \frac{1}{2}R^{-1}\Omega^{-\frac{1}{3}}\omega^{\frac{1}{3}}\gamma_S\ \dot{\epsilon}_{cr}\dot{\epsilon}_{cr} - C\mu U\Omega^{\frac{2}{3}}\,L^{-1}\left\{\frac{\frac{4}{3}ln\,\omega\,\omega^{\frac{1}{3}} - \omega^{\frac{1}{3}}}{(ln\,\omega)^2}\right\}\dot{\omega}\dot{\epsilon}_{cr} \\
&= \ddot{\phi}_d(\epsilon_{cr}, \dot{\epsilon}_{cr}) + \frac{1}{2}R^{-1}\Omega^{-\frac{1}{3}}\omega^{\frac{1}{3}}\gamma_S\ \dot{\epsilon}_{cr}\dot{\epsilon}_{cr} - C\mu\ U\Omega^{\frac{2}{3}}\,L^{-1}\left\{\frac{\frac{4}{3}ln\,\omega\,\omega^{\frac{1}{3}} - \omega^{\frac{1}{3}}}{(ln\,\omega)^2}\right\}\frac{3}{2}\omega\dot{\epsilon}_{cr}\dot{\epsilon}_{cr} \qquad \text{(via Eq. S4)} \\
&= \ddot{\phi}_d(\epsilon_{cr}, \dot{\epsilon}_{cr}) + \frac{1}{2}R^{-1}\Omega^{-\frac{1}{3}}\omega^{\frac{1}{3}}\gamma_S\ \dot{\epsilon}_{cr}\dot{\epsilon}_{cr} - 2C\mu U\Omega^{\frac{2}{3}}\,L^{-1}\frac{\omega^{\frac{4}{3}}}{\ln\omega}\dot{\epsilon}_{cr}\dot{\epsilon}_{cr}
\end{aligned}$$

Now multiplying throughout by $\Omega^{\frac{1}{3}}\omega^{-\frac{1}{3}}$ and expanding the dissipation function using Taylor series in ϵ_{cr} we get:

$$\ddot{g} = \left\{\frac{\partial^2\phi_d(\dot{\epsilon}_{cr}, \epsilon_{cr})}{\partial\epsilon_{cr}^2}\ \Omega^{\frac{1}{3}}\omega^{-\frac{1}{3}} + \frac{\gamma_S}{2R} - \frac{C'\mu U}{L}\ \Omega\frac{\omega}{\ln\omega}\right\}\ \dot{\epsilon}_{cr}\dot{\epsilon}_{cr} \tag{S8}$$

Now recalling that $\omega, \Omega \gg 1$ and from Eq. S3 the vanishing second derivative gives us the following condition for instability at critical point:

$$\left(\frac{\partial^2\phi_d(\dot{\epsilon}_{cr}, \epsilon_{cr})}{\partial\epsilon_{cr}^2}\right)_c e^{-\frac{\epsilon_{cr,c}}{2}} + \frac{1}{2}\frac{\gamma_S}{R} - \frac{C'\mu U}{L}\ \frac{\Omega^2}{\ln\Omega}e^{\frac{3\epsilon_{cr,c}}{2}} = 0 \tag{S9}$$

Supplementary Videos

Supplementary Video 1: Video shows failure of a *P. fluorescens* streamer. Video is real-time and tracking of two Lagrangian points (‘1’ & ‘2’) are shown.
